# Cases of Impaired Oxidative Burst in HIV-Exposed Uninfected Infants’ Neutrophils—A Pilot Study

**DOI:** 10.3389/fimmu.2017.00262

**Published:** 2017-03-09

**Authors:** Anicet Christel Maloupazoa Siawaya, Amandine Mveang-Nzoghe, Ofilia Mvoundza Ndjindji, Armel Mintsa Ndong, Paulin N. Essone, Joel Fleury Djoba Siawaya

**Affiliations:** ^1^Unités de Recherche et de Diagnostics Spécialisés, Laboratoire National de Santé Publique, Libreville, Gabon; ^2^Unité de Virologie, Laboratoire National de Santé Publique, Libreville, Gabon; ^3^Faculty of Health Sciences, Institute of Infectious Diseases and Molecular Medicine (IDM), Division of Immunology and South African Medical Research Council (SAMRC) Immunology of Infectious Diseases, University of Cape Town, Cape Town, South Africa

**Keywords:** HIV, neutrophils, reactive oxygen species, nitroblue tetrazolium, infants

## Abstract

An increased risk of serious bacterial infections in HIV-exposed uninfected (HEU) infants has been demonstrated. Although neutrophils are essential for the protection of infants against bacterial infections, no study has investigated their profile in HEU infants to date. In this study, we assessed the function of neutrophils in HEU infants using the nitroblue tetrazolium reduction test. Among 25 HEU infants, 9 (36%) showed a reduced ability of their neutrophils to produce reactive oxygen species upon stimulation with bacteria. No alteration of total neutrophil counts was noted in the blood of HEU infants indicating that the alteration observed in the 36% of HEU infants may only be functional. Conclusively, impaired neutrophil function could be a factor of vulnerability in HEU infants.

## Introduction

The implementation of mother-to-child transmission prevention programs worldwide is arguably one of the major public health successes of the present century. Indeed, the proposed recommendations on the antiretroviral therapy on mothers and their infants, together with improved obstetric management and the updated protocols on breastfeeding from HIV-infected mothers have considerably reduced the number of infected infants in developing countries (1, 2).

Currently, it is estimated that HIV-exposed uninfected (HEU) infants represent up to 30% of all births in Africa (3, 4). Although mother-to-child transmission prevention programs have dramatically reduced the mother-to-child transmission rate, HEU infants are still more vulnerable to infections when compared to HIV-unexposed (HU) infants from HIV-uninfected mothers. This was robustly demonstrated in a study conducted in Zimbabwe between 1987 and 2000, where more than 14,000 participants were enrolled (5). In this study, an elevated mortality rate of HEU infants was observed (5). The 2-year mortality rate was three times higher in the HEU cohort when compared to the HU cohort (5, 6). Morbidity and mortality were at their peak levels in the 3–6 months old children, and the majority of the deaths were associated to lower respiratory tract infections. This seminal observation has been supported by several other studies in Africa that have independently reported a more vulnerable immune system thus a higher risk of infection of HEU infants when compared to HU infants (7–13).

Intensive research on HIV and antiretroviral (ARV)-drug therapy has improved our understanding of the immune susceptibility of HIV-infected population (14–16); however, our understanding of HIV/therapy exposure on HEU infants is still limited. A number of studies have reported a lower numbers of naive CD4^+^ cells, a reduced thymic output (17) and an impaired humoral response to vaccines (18) in HEU infants. However, the vulnerability of the immune system of HEU infants in the context of the alteration of the production of reactive oxygen species (ROS), a class of signaling molecules that regulate many signal-transduction pathways and play critical roles in cell survival, death, and immune defenses during infection (15, 19) has never been addressed.

Neutrophils are major producers of ROS and essential effector cells of the innate immune response against invading pathogens and the pivots of innate immunity; as they are first and powerful defense system against invading pathogens (20). Microbicides and cytotoxic activities of neutrophils depend on very intricate mechanisms including the release of proteolytic enzymes and the rapid production of ROS (oxidative burst) (21). The respiratory burst is the major mechanism by which neutrophils kill engulfed bacteria (22). In the light of the foregoing and of the observed susceptibility of HEU infants to infections, we investigated the phagocytes oxidative capacity of HEU infants.

## Materials and Methods

### Participants

Twenty-five healthy HEU infants aged 6- to 12-week olds visiting the National Laboratory of Public Health in Libreville (Gabon) were prospectively recruited from March to August 2015. The mothers and infants information on preventive treatment, breastfeeding, time of anti-viral therapy initiation were recorded.

HIV perinatal infection (RT-PCR Cobas, Biomerieux, France) were checked from each HEU infant in peripheral blood at 1.5, 3, 6, and 9 months after birth as part of their scheduled checked up in the reference laboratory in Gabon. An additional sample was taken to determine anti-HIV-1 antibodies seroconversion by ELISA at 18 months old. All HIV-positive infants and infants with any clinical condition at the time of recruitment were excluded from the study.

### Ethics Statement

Informed consent was obtained from parents of all study participants. The National Laboratory of Public Health Ethics Review Board of Gabon approved the protocol. We adhered to the World Medical Association’s Declaration of Helsinki and Good Clinical Practice guidelines during the treatment of all participants and handling of their personal data.

### Neutrophils Oxidative Burst Response Assay

The neutrophils oxidative burst response was performed on blood collected from 6- or 12-week-old infants using the *In vitro* diagnostic quality Sigma-Aldrich nitroblue tetrazolium (NBT) reduction test (St. Louis, MO, USA). The test was done according to the manufacturer protocol. Briefly, venous blood from infants was drawn into a heparin tube. Heparinized blood (0.05–0.2 ml) was mixed with the NBT mixture (1–1.2 ml) and 5 μl of stimulant (bacterial extracts/endotoxins) in the stimulated samples. Fifty microliters of heparinized blood–NBT mixture were transferred onto a clean glass slide to make a moderately thick smear. The smears were treated with 1 ml of ACCUSTAIN Wright Stain for 15 s and flooded with 1 ml of distilled water for 30 s, rinsed and air-dried. Three readers evaluated the percentage of formazan-containing neutrophils on stained slides under a microscope.

### Interpretation NBT Reduction Test Results

According to the literature, 69–100% of resting neutrophils are NBT-positive in healthy newborn infants (23). In the present study, we set the threshold for the diagnosis of oxidative burst defect at 60% of neutrophils reducing NBT after bacterial extracts stimulation. Therefore, neutrophils function was defined as normal if more than 60% of neutrophils reduced NBT after bacterial extracts stimulation. Impaired or reduced ROS production was evoked when a subject shows less than 60% of neutrophils reducing NBT after bacterial extracts stimulation.

### Statistical Analysis

All statistical analyses were done using the software GraphPad Prism version 6. Descriptive statistics and odds ratios were used to characterize the study population. The difference between infants with neutrophils dysfunction and infants with functional neutrophils was assessed using the Mann–Whitney *U*-test (significance set *p*-value ≤0.05).

## Results and Discussion

Infections in infants are an important cause of morbidity and mortality. Understanding their immune system is crucial to develop new approaches to fight and prevent diseases and improve their health. Because in their early life, infants depend mainly on their innate immune system for protection against infections, we studied HEU infants’ neutrophils function.

**Figure 1:**
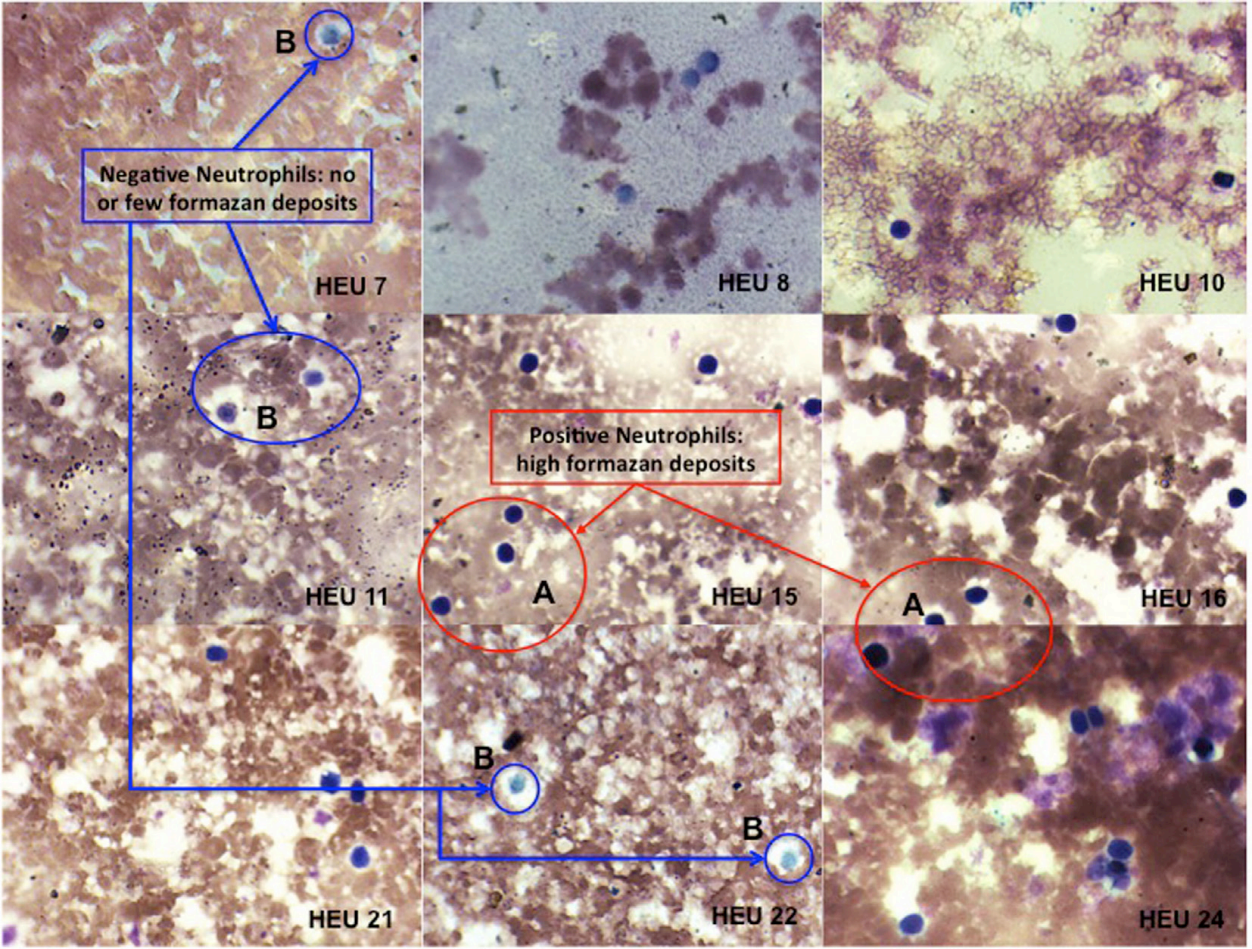
**Photomicrographs of nitroblue tetrazolium (NBT) dye reduction by blood neutrophils from HEU infants (at ×100 magnification)**. Positive = neutrophils contain increased formazan deposits visible as a large dark purple to black intra-cytoplasmic inclusions **(A)**. Negative = neutrophils contain few or no formazan deposits **(B)**.

**Figure 2:**
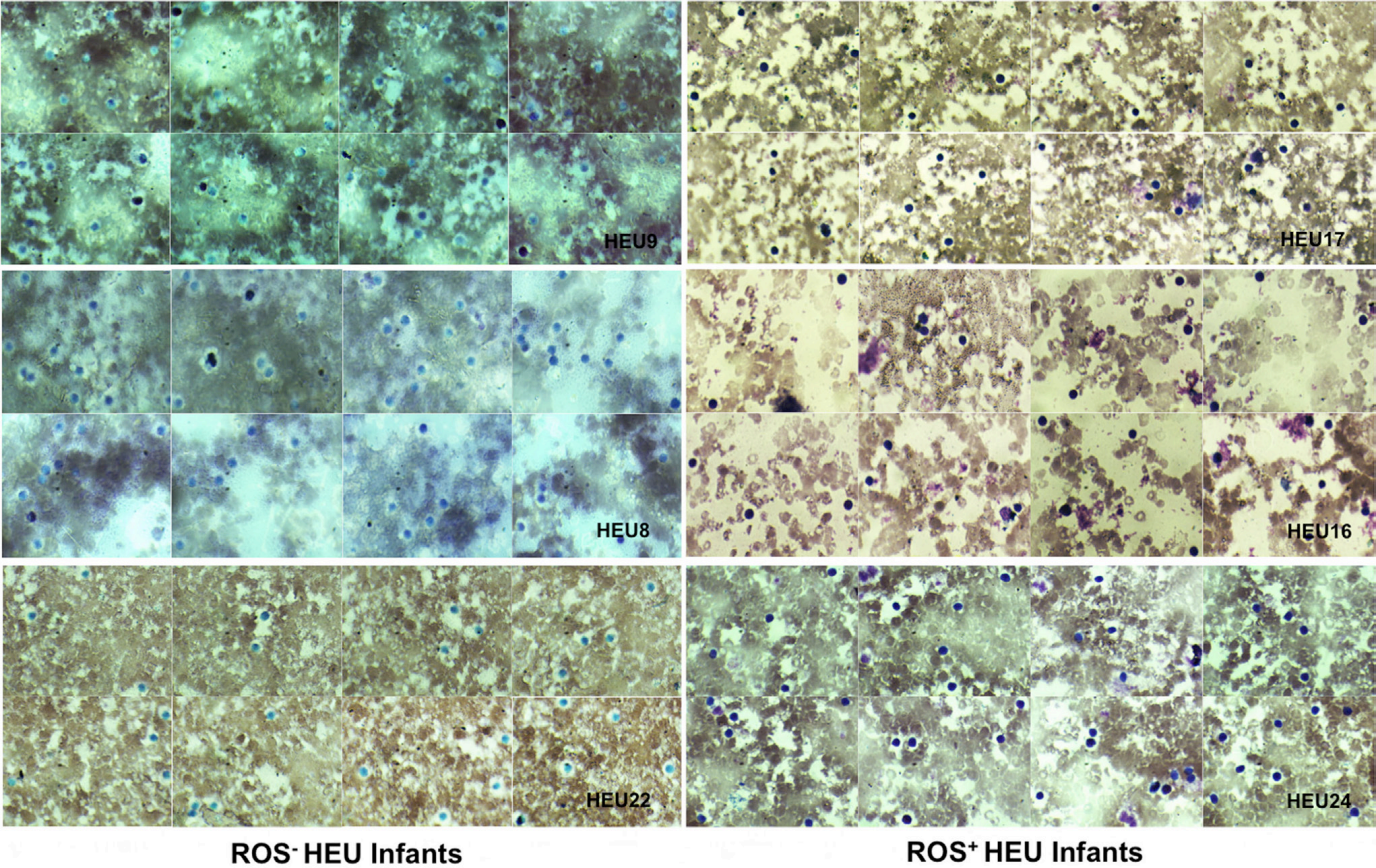
**Photomicrographs of nitroblue tetrazolium (NBT) dye reduction profiles of selected HIV-exposed uninfected (HEU) infants blood neutrophils (at ×100 magnification)**. Reactive oxygen species (ROS)^−^ HEU infants indicate infants with impaired ROS production. ROS^+^ HEU infants indicate infants with normal ROS production. ROS^+^ HEU infants illustrated by HEU 17, 16, and 27 showed increased formazan deposits visible as a large dark purple to black intra-cytoplasmic inclusions whereas, ROS^−^ HEU infants illustrated by HEU 8, 9, and 22 showed few or no formazan deposits.

Neutrophils oxidative burst was assessed for 25 HIV-exposed infants referred to our study site; The National Laboratory of Public Health (Libreville, Gabon). This study focused in HEU infants as the HU infants are not follow-up in our study site. It was therefore difficult to include HU children in this pilot study. Nevertheless, we manage to include three HU infants in the study. Their results are included in the study as technical control. The limited number of HU (3 HU infants) obtained in this study was not enough for experimental control. Their immune responses were not directly compared to HEU immune response. ROS are crucially involved in microbial killing (22, 24). Figure 1 illustrates the quantification of NBT-test negative and positive neutrophils. Seventeen of the twenty-five infants were males and eight were females as shown in the Table 1. Sixteen HIV-exposed infants (64%) had normal neutrophils profile after antigen stimulation. In those infants, the rate of neutrophils positive for formazan deposits (reduced NBT) after bacterial extracts stimulation ranged from 67 to 97.7% (Table 1). We observed a high number of resting neutrophils producing ROS (reducing NBT) in most HEU infants with functioning neutrophils. This observation was not surprising, as an increased reduction of NBT by neutrophils of newborn infants has already been reported (23, 25). Tovo and Ponzone illustrated the presence of humoral factors stimulating ROS secretion by neutrophils leading to positive NBT reduction (25) in the plasma of neonates. The high percentages of NBT positive cells could also be explained by *in utero* exposure to a pro-inflammatory fetal environment (26). Figure 2 shows selected HEU infants ROS secretion profile after antigen stimulation.

**Table 1:** **Nitroblue tetrazolium (NBT)-test results and clinical information**.

Infant ID	Age (in weeks)	Gender	NBT-positive neutrophils		Interpretation	Infants preventive Thx	Breastfeeding	Mother start of antiretroviral therapy		
							
			Non-stimulated (%)	Stimulated (%)				Before pregnancy	During pregnancy	Therapy
HEU1	10	Male	86.7	88	*Normal neutrophils*	Nevirapine/co-trimoxazole	No	No	Yes @ 6 months	EFV-TDF-LVD
HEU2	6	Male	60	89	*Normal neutrophils*	Nevirapine/co-trimoxazole	No	Yes	No	TDF-3TC-EFV
HEU3	12	Female	75	85	*Normal neutrophils*	Zidovidine/co-trimoxazole	No	Yes	No	3TC-Zido-EFV
HEU4	6	Female	73	85	*Normal neutrophils*			Yes	No	TDF-3TC-EFV
HEU5	12	Female	47	92.7	*Normal neutrophils*	Zidovidine/co-trimoxazole	No	Yes	No	
HEU6	6	Male	78	91	*Normal neutrophils*	Nevirapine/co-trimoxazole	No	Yes	No	
**HEU7**	**6**	**Female**	**9.3**	**12.7**	***Impaired reactive oxygen species (ROS) production***	**Nevirapine/co-trimoxazole**	**No**	**No**	**No**	**None**
**HEU8**	**10**	**Male**	**8.7**	**4.7**	***Impaired ROS production***	**Nevirapine/co-trimoxazole**	**No**	**No**	**Yes @ 4 months**	**TDF-FTC-EFV**
**HEU9**	**10**	**Male**	**9.5**	**7**	***Impaired ROS production***	**Nevirapine/co-trimoxazole**	**No**	**No**	**Yes @ 4 months**	**TDF-FTC-EFV**
**HEU10**	**6**	**Male**	**14**	**35**	***Impaired ROS production***	**Nevirapine**	**No**	**Yes**	**NO**	
**HEU11**	**6**	**Female**	**48.3**	**38**	***Impaired ROS production***	**Nevirapine/co-trimoxazole**	**No**	**No**	**Yes @ 6 months**	
**HEU12**	**6**	**Male**	**37**	**34**	***Impaired ROS production***	**Nevirapine/co-trimoxazole**	**No**	**No**	**Yes @ 2 months**	
HEU13	6	Female	6.3	97.7	*Normal neutrophils*	Nevirapine/co-trimoxazole	No	Yes		ABC-3TC-LPV-RTV
**HEU14**	**12**	**Male**	**14**	**23**	***Impaired ROS production***	**Nevirapine/co-trimoxazole**	**Yes (for 3 months)**	**No**	**Yes @ 6 months**	**TDF-3TC-EFV**
HEU15	6	Male	93.7	94.7	*Normal neutrophils*	NO	Yes	No	Yes @ 3 months	TDF-FTC-EFV
HEU16	6	Male	91.7	98	*Normal neutrophils*	Nevirapine/co-trimoxazole	No	Yes	No	
HEU17	8	Male	96	99	*Normal neutrophils*	Nevirapine	No	No	Yes @ 3 months	TDF-FTC-EFV
HEU18	7	Male	75	81.3	*Normal neutrophils*	Nevirapine/co-trimoxazole	No		Yes @ 4 months	
HEU19	6	Female	74.7	73.3	*Normal neutrophils*	Nevirapine/co-trimoxazole	No	No	Yes @ 4 months	
HEU20	6	Male	86	88	*Normal neutrophils*	Nevirapine/co-trimoxazole	No	Yes	No	
**HEU21**	**6**	**Female**	**33.3**	**30.5**	***Impaired ROS production***	**Nevirapine/co-trimoxazole**	**No**	**Yes**	**No**	
**HEU22**	**6**	**Male**	**3**	**3**	***Impaired ROS production***	**Nevirapine/co-trimoxazole**	**No**	**Yes**	**No**	**TDF-3TC-EFV**
HEU23	6	Male	74.7	67	*Normal neutrophils*	Nevirapine/co-trimoxazole	No	Yes	No	
HEU24	6	Male	67	80	*Normal neutrophils*	Nevirapine/co-trimoxazole	No	Yes	No	AZT-3TC-NVP
HEU25	6	Male	74	83.7	*Normal neutrophils*	Nevirapine/co-trimoxazole	No	No	Yes @ 7 months	AZT-3TC-NVP
HU1	12	Female	56	72.7	*Normal neutrophils*	NA	NA	NA	NA	NA
HU2	8	Female	53	92	*Normal neutrophils*	NA	NA	NA	NA	NA
HU3	6	Male	76.3	95.3	*Normal neutrophils*	NA	NA	NA	NA	NA

*Bold font indicates Impaired ROS production*.

Nine HIV-exposed infants (36%) showed impaired ROS production after stimulation with bacterial extracts (Table 1). In those infants, the rate of neutrophils positive for formazan deposits after bacterial extracts stimulation ranged from 3 to 35%. The ability of neutrophil to produce ROS after exposure to bacteria or bacteria extracts indicates the functionality of these immune cells. Several studies have addressed the alteration of the neutrophil compartment in HEU infants. A telling example is that of an European collaborative study which reported that exposure to ARV does not affects HEU children neutrophils count during the first months of life (27). This is consistent with our present data and further reinforces the notion that neutrophil counts might not be a reliable early indicator of the defective immune system of HEU infants.

Our study does not only assess the number of neutrophils but beyond the quantitative appraisal of these important innate immune effector cells, also looks at their functionality in the form of ROS secretion. We report an impaired production of ROS by neutrophils of 36% of HEU infants suggestive of an impaired immune system in these infants. This finding is clearly important but would urgently require to be validated in a larger study. Given the small number, the primary results presented in this study could be a call to the scientific community to further investigate early marker(s) of immune dysfunction in HEU infants.

The metabolic defect observed in a number of HEU infants could find its explanations by the transfer of immunosuppressive viral products across the placenta that has been suggested to suppress infants’ immune system without productive infection (4). Moreover, antenatal ARV-prophylaxis therapy for the prevention of mother-to-child transmission has been reported to be toxic to mitochondria and to modulate the immune system of HEU infants (4). This was further suggested experimentally by Michailidis et al., who showed that neutrophil oxidative burst was significantly lower in HIV-patients receiving highly active anti-retroviral treatment (HAART) when compared to treatment-naïve patients, supporting the plausible link between HAART and neutrophils impaired oxidative burst (28). We now provide additional evidences for such a link as our contingency table analysis showed that infants born from mothers who started ARV-therapy during pregnancy had an odds ratio of 2.8 for oxidative burst defect when compared to infants born from mothers who started ARV-therapy before pregnancy. Although suggestive, our present findings need to be validated in larger studies for dispositive answers on the role of ARV and/or HIV *in utero* HIV exposure on the neutrophil functions and the relationship of this apparent interdependence on the impaired immune system of HEU infants.

We believe that the observed impaired function of neutrophils in HEU infants to be transient. In fact, a couple of HEU infants who had impaired neutrophils function and that was brought for an unscheduled visit 2 months after the recruitment had recovered neutrophils function (data not shown). This is not surprising as Le Chenadec et al. reported that ARV exposure had a transient effect on several selected hematological variables in HEU infants (29).

**Figure 3:**
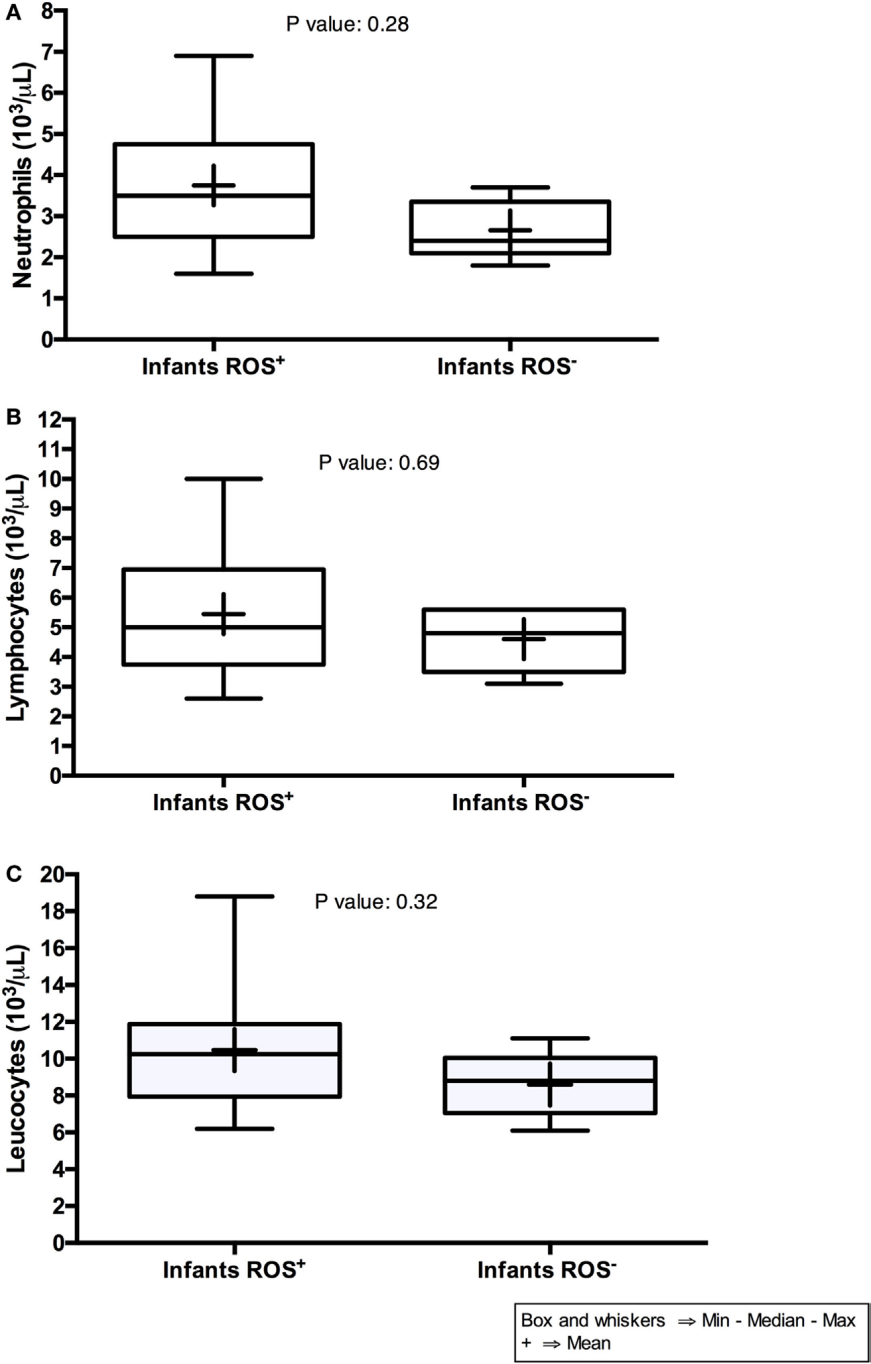
**Neutrophils, lymphocytes, and leukocytes counts of HIV-exposed uninfected infants**. On the graphs, *Infants* (*ROS^+^*) indicate infants with normal reactive oxygen species (ROS) production, whereas *Infants* (*ROS*^−^) indicate infants with impaired ROS production.

Comparing the blood of infants with dysfunction neutrophil function with that of infants with unaltered responsiveness, we found no significant differences in their total numbers of neutrophils, lymphocytes, and leukocytes (Figure 3) further re-emphasizing the poor potential of assessing total immune cell numbers to predict the vulnerability of the immune system of HEU infants.

## Conclusion

The present study contributes to the global effort to understand the immune determinants of HEU infants increased susceptibility to infections during the first month of their life. Using NBT reduction assay, we found impaired oxidative burst in 36% of HEU infants evaluated with no alteration in neutrophil counts suggesting that impaired neutrophils function could be one of the immune dysfunctions found in a number of HEU infants. The present results need to be validated in a larger study.

## Author Contributions

AS, AM-N, and ON: recruited participant, performed research, and analyzed data. AN: contributed to provide analytical tools. PE: designed research and proofread the paper. JS: designed the research, performed research, contributed vital reagents and analytical tools, analyzed data, and wrote the paper.

## Conflict of Interest Statement

The authors declare that they have no competing interests. Also no financial or other competing interests exist.
